# Marked regional endothelial dysfunction in mottled skin area in patients with severe infections

**DOI:** 10.1186/s13054-017-1742-x

**Published:** 2017-06-23

**Authors:** Simon Bourcier, Jérémie Joffre, Vincent Dubée, Gabriel Preda, Jean-Luc Baudel, Naïke Bigé, Guillaume Leblanc, Bernard I. Levy, Bertrand Guidet, Eric Maury, Hafid Ait-Oufella

**Affiliations:** 1 Assistance Publique – Hôpitaux de Paris (AP-HP), Hôpital Saint-Antoine, Service de réanimation médicale, 184 rue du Faubourg Saint-Antoine, 75571 Paris, Cedex 12 France; 2 Université Pierre-et-Marie Curie, Paris 6, France; 3 Inserm U1136, Paris, F-75012 France; 4 Inserm U970, Centre de Recherche Cardiovasculaire de Paris (PARCC), Paris, France; 5 Department of Anesthesiology and Critical Care Medicine, Faculty of Medicine, Université Laval, Québec, QC Canada

**Keywords:** Infection, Mottling, Tissue perfusion, Mortality, Endothelial function

## Abstract

**Background:**

Mottling around the knee, reflecting a reduced skin blood flow, is predictive of mortality in patients with septic shock. However, the causative pathophysiology of mottling remains unknown. We hypothesized that the cutaneous hypoperfusion observed in the mottled area is related to regional endothelial dysfunction.

**Methods:**

This was a prospective, observational study in a medical ICU in a tertiary teaching hospital. Consecutive adult patients with sepsis admitted to ICU were included. After resuscitation, endothelium-dependent vasodilation in the skin circulation was measured before and after iontophoresis of acetylcholine (Ach) in the forearm and the knee area. We analyzed the patterns of induced vasodilatation according to the presence or absence of mottling and vital status at 14 days.

**Results:**

We evaluated 37 septic patients, including 11 without and 26 with septic shock. Overall 14-day mortality was 22%. Ten patients had mottling around the knee (10/37, 27%). In the knee area, the increased skin blood flow following iontophoresis of Ach was lower in patients with mottled skin as compared to patients without mottled skin (area under curve (AUC) 3280 (2643–6440) vs. 7980 (4233–19,707), both *P* < 0.05). In the forearm area, the increased skin blood flow following iontophoresis of Ach was similar in patients with and without mottled skin. Among patients with septic shock, the increased skin blood flow following iontophoresis of Ach in the knee area was significantly lower in non-survivors as compared to survivors at 14 days (AUC 3256 (2600–4426) vs. 7704 (4539–15,011), *P* < 0.01). In patients with septic shock, the increased skin blood flow in the forearm area following iontophoresis of Ach was similar in survivors and non-survivors at 14 days.

**Conclusion:**

Mottling is associated with regional endothelial dysfunction in patients with septic shock. Endothelial dysfunction in the knee skin area was more pronounced in non-survivors than in survivors.

**Electronic supplementary material:**

The online version of this article (doi:10.1186/s13054-017-1742-x) contains supplementary material, which is available to authorized users.

## Background

Sepsis is a common and life-threatening condition that develops in response to bacterial injury, leading to tissue hypoperfusion and multi-organ damage. The reduction of tissue perfusion is mainly due to microcirculatory abnormalities detectable at the onset of sepsis [1]. The severity [2, 3] and persistence [4] of these microvascular abnormalities are closely correlated with the patient’s prognosis. During septic shock, intra-vital microscopy in animals and humans has identified alterations in the microcirculation, with heterogeneous perfusion within each organ [5].

Mottling, defined as patchy skin discoloration, reflects reduced skin blood flow [6] and low tissue oxygen saturation [7] and has been suggested as a tool for clinical evaluation of tissue perfusion in patients with severe infection [8]. Extensive mottling extension has been demonstrated to be predictive of mortality in patients with septic shock, independently of systemic haemodynamic parameters [3, 9, 10]. However, the mechanism responsible for the specific regional reduction of blood flow in the mottled skin area remains unknown. Direct capillary obstruction by platelet aggregation and coagulation cascade activation has been suggested as a mechanism responsible for skin hypoperfusion [11] and has been documented in patients with meningococcemia [12]. However, in the absence of diffuse intravascular coagulation, the widely accepted concept is vasoconstriction mediated by major sympathetic neuroactivation [13].

To date, endothelial dysfunction has been indirectly assessed by the measurement of various inflammatory markers released in the plasma by the vascular endothelium [14]. However, these circulating markers cannot be used to assess regional or organ-specific perfusion and endothelial dysfunction. Regional endothelial function can be assessed by the ability of blood vessels to vasodilate in response to endothelial nitric oxide (NO) production stimulated by pharmacological stimuli or shear stress [15]. Endothelial dysfunction has been previously reported during sepsis [16] and septic shock [17] using large artery flow-mediated vasodilatation (FMD) measured by ultrasonography. Another recent method is applied to the skin microcirculation. Endothelium-dependent vasodilation of skin resistance vessels can be induced by transdermal iontophoretic application of acetylcholine (Ach). Transdermal iontophoresis of Ach induces NO production by endothelial cells, inducing relaxation and vasodilation of smooth muscle cells. The resulting local increase in blood flow can be subsequently measured by laser Doppler flowmetry [18, 19].

The aim of this study was to assess microcirculatory endothelial skin function using transdermal iontophoresis of Ach in mottled and non-mottled areas of the knee and the forearm area in patients with severe infection, and to assess the relationship between endothelial dysfunction and the outcome at day 14. We hypothesized that the cutaneous hypoperfusion observed in the mottled area is associated with local impairment of vasomotor regulation due to endothelial dysfunction and is associated with an adverse outcome at 14 days.

## Methods

We conducted a prospective, observational study in an 18-bed intensive care unit (ICU) in a tertiary teaching hospital in France. We included consecutive adult patients (≥18 years of age) admitted to the ICU for sepsis, with or without septic shock, according to the third international consensus definitions for sepsis and septic shock [20]. Patients could be admitted from the emergency department or the medical wards. Patients with sepsis were included at ICU admission and patients with septic shock were included when vasopressors were required (within 3 h of admission). The timing of vasopressor initiation was defined as zero hours (H0) in patients with septic shock. Patients with dark skin were excluded because of the impossibility of assessing the mottling score.

### Protocol for the management of patients

Management of the included patients was guided by our local protocol, adapted from international guidelines [21]. In patients with septic shock, intravenous volume expansion was provided to achieve predefined endpoints: pulse pressure variation <13% [22], no response to passive leg raising [23], or no respiratory variations of the inferior vena cava diameter (assessed by echography) [24]. Norepinephrine was used in a stepwise manner to achieve predefined endpoints: mean arterial pressure (MAP) ≥65 mmHg and urine output ≥0.5 mL/Kg/h. All patients were investigated with transthoracic echocardiography (Vivid 7 dimension’06, GE Healthcare^®^). If cardiac dysfunction was identified (left ejection fraction <30% by Simpson bi-plan methodology), inotropic therapy was introduced and/or epinephrine was used to replace norepinephrine. Mechanical ventilation was provided when needed. If required, patients were sedated with propofol and/or midazolam and analgesia was provided with sufentanil. Glycemic control and venous thrombosis prophylaxis were provided.

### Assessment of endothelial function in the skin microcirculation

The endothelial function in the skin microcirculation was measured in two different skin areas; the first one in the forearm, which is an area where mottling does not classically develop and the second one in the knee where mottling predominates. The assessment of endothelial function in the skin microcirculation was performed using transdermal iontophoresis of Ach. This non-invasive technique allows for the local transfer of charged substances (Ach) across the skin by the use of a weak electrical current (Additional file 1).

Following application of a weak current, electrical potential difference will actively cause ions and molecules bearing a net electrical charge to migrate in solution. The direction and speed of migration can be achieved by adjusting the polarity and magnitude. The total amount of acetylcholine delivered into the skin is related to the current and the duration of application (i.e., the electrical charge). The iontophoresis drug delivery chamber was attached to the flexor surface of the non-dominant forearm and in the middle of the knee area. The positive lead of the current source was attached to the drug delivery chamber, and the negative lead (i.e., reference electrode) to a conductive hydrogel pad installed on the wrist. After measurement of baseline blood flow for 60 seconds, three successive applications of Ach were made, every 60 seconds, using anodal current (0.12 mA for 12 seconds each). The drug delivery chamber was loaded with 80 μL of Ach (pilocarpine 2% doses) [19].

The response of skin blood flow to Ach iontophoresis was assessed by the laser Doppler flowmetry technique. The laser light penetrates the skin and is partially backscattered by red blood cells. According to the Fizeau-Döppler principle, the frequency of the backscattered light was changed in proportion to the velocity of the red blood cells. The frequency shifts are converted into a voltage signal that is proportional to the number and velocity of the illuminated red blood cells. A lLaser Doppler flowmeter probe (Periflux 5000, Perimed) embedded within a heating drug delivery chamber was used in combination with a current-controlled delivering device (PeriIont, Perimed). Laser Doppler flowmeter signals were recorded continuously using an interfaced computer with acquisition software (Perisoft, Perimed). Skin blood flow was recorded during a 10-minute period after the first iontophoresis of Ach. Measurements of skin blood flow were quantified as the maximal increase (peak value) and the area under the curve (AUC) (Additional file 2). The analysis of skin endothelial function was performed by independently by a physician who did not participate in patient care.

### Data collection

The following general characteristics of the patients were recorded: age, sex, previous chronic illness, severity of illness evaluated by the Sequential Organ Failure Assessment score (SOFA score) 6 h after inclusion [25] and the Simplified Acute Physiologic Score II (SAPS II) [26], primary site of infection, mode of ventilation, and vasopressor use. We collected global hemodynamic parameters: mean arterial pressure (MAP), heart rate (HR) and cardiac index (CI), and microcirculatory dysfunction, and organ perfusion parameters: arterial lactate level, urine output, and mottling score at 6 h after inclusion.

### Statistical analysis

Patient characteristics were expressed as median (25^th^–75^th^ percentiles) or number and percentage, as appropriate. Data were first analyzed according to the presence or absence of mottled skin in the knee area and then according to three different groups of patients; patients with sepsis, patients with septic shock who were still alive at 14 days (survivors) and patients with septic shock who had died by 14 days (non-survivors). Differences among groups were assessed using the Kruskal-Wallis test with post hoc Mann-Whitney analysis. The measurements obtained in the three groups were compared with one-way analysis of variance (ANOVA). When the *P* value was significant, pairwise comparisons were carried out using modified *t* tests. All tests were computed with the R software (R Foundation for Statistical Computing). Significance was defined as a two-sided *P* value <0.05.

The protocol was approved by our institution’s ethical committee, Comité de Protection des Personnes (CPP Saint-Louis, Paris, France). This was a non-invasive observational study without any specific intervention according to the Ach iontophoresis result. All patients and families were informed that anonymous data could be used for academic research and gave their consent.

## Results

### Baseline characteristics of patients

Thirty-seven consecutive adult patients admitted to the ICU for severe infections were included in the study (Additional file 3). There were 11 patients with sepsis (11/37, 30%) and 26 patients with septic shock (26/37, 70%). The overall mortality at 14 days was 22% (8/37). The most prevalent primary sites of infection were the lungs (52%) and the abdomen (29%). Patients with septic shock had higher SAPS II and SOFA scores and more frequently required invasive therapy (such as mechanical ventilation) than patients with sepsis (Table 1). All patients with septic shock received norepinephrine (at H6, median dose 0.30 (0.15–0.60) μg/Kg/min) but none received dobutamine.

**Table 1:** Hemodynamic and tissue perfusion parameters of patients

Hemodynamic parameters at H6	Sepsis	Septic shock survivors	Septic shock non-survivors	*P* value
Number	11	18	8	-
SAPS II	38 (30; 41)	53 (35; 70)	80 (53; 82)	a, <0.001 b, 0.08
SOFA score	5 (3; 6)	9 (7; 13)	16 (12; 17)	a, <0.001 b, <0.001
Mechanical ventilation (%)	12	55	75	a, 0.03 b, ns
Norepinephrine dose (μg/kg/min)	-	0.30 (0.13; 80)	1.10 (0.60; 1.50)	b, 0.009
MAP (mmHg)	75 (66; 83)	73 (68; 79)	68 (65; 76)	a, ns b, ns
Cardiac index (L/min/m^2^)	2.7 (2.4; 3.0)	2.6 (2.4; 3.4)	2.5 (2.0; 3.1)	a, ns b, ns
Urinary output (mL/Kg/h)	1.0 (0.7; 2.1)	0.9 (0.5; 1.3)	0.4 (0.1; 0.5)	a, ns b, 0.02
Lactate level (mmol/L)	1.3 (1.0; 1.8)	1.6 (0.9; 2.5)	5.3 (3.0; 9.5)	a, ns b, 0.004
Mottling presence, *n* (%)	1 (9)	5 (27)	4 (50)	a, ns b, ns
Temperature, core (°C)	37.7 (36.3; 38.7)	38.1 (37.8; 39.3)	36.8 (35.4; 37.9)	a, ns b, ns
Temperature, forearm skin (°C)	30.3 (28.9; 31.7)	30.1 (28.7; 31.6)	29.1 (27.7; 29.9)	a, ns b, ns
Temperature, knee skin (°C)	30.4 (28.9; 31.6)	29.4 (28.3; 30.1)	28.9 (27.1; 29.9)	a, ns b, ns

Data are expressed as number (percentage) or median (interquartile range). Statistical analysis: *a*, sepsis vs. septic shock survivors; *b*, septic shock survivors vs. septic shock non-survivors. *H6* 6 h after initition of vasopressors, *SOFA* Sequential Organ Failure Assessment; *SAPS II* Simplified Acute Physiology Score; *MAP* mean arterial pressure; *ns* non significant

### Assessment of hemodynamic parameters

Hemodynamic parameters 6 h after initial resuscitation in patients with sepsis, septic shock survivors and septic shock non-survivors are presented in Table 1. Mean arterial blood pressure and cardiac index were similar between groups. Tissue perfusion parameters were similar in patients with sepsis and septic shock survivors. However, when compared to surviving patients with septic shock, non-surviving patients with septic shock had higher arterial lactate (5.3 (3.0–9.5) vs. 1.6 (0.9–2.5) mmol/L, *P* = 0.004) and lower urine output (0.4 (0.1–0.5) vs. 0.9 (0.5–1.3) mL/Kg/h, *P* = 0.02). Ten patients had mottling around the knee (10/37, 27%). Mottling was more frequently observed in non-survivors as compared to survivors, but the difference was not statistically significant.

We compared hemodynamic parameters in sepsis/septic shock patients according to the presence of mottling at H6 (Additional file 4). We did not observe any difference between groups except for norepinephrine dosage that was significantly higher in patients with knee mottling.

### Baseline microcirculatory skin blood flow assessment

**Fig. 1:**
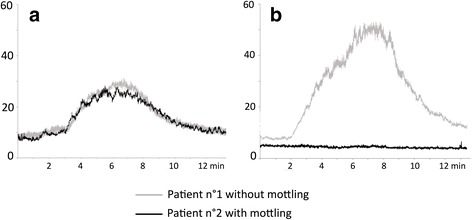
Example of skin microcirculatory blood flow assessment in a patient without mottling (*Patient n°1*) and in a patient who had mottling in the knee area (*Patient n°2*). Skin blood at baseline and after iontophoresis acetylcholine was measured on the forearm area (**a**) and in the knee area (**b**)

**Fig. 2:**
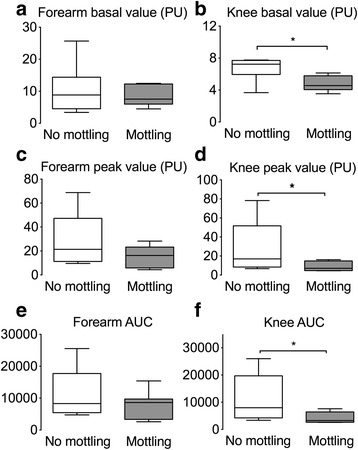
Analysis of skin microcirculatory blood flow in patients with knee mottling (n = 10) and in patients without knee mottling (n = 27) at 6 h. Skin blood flow was assessed on the forearm and the knee areas at baseline (**a**, **b**), and after acetylcholine iontophoresis (peak value (**c** and **d**) and area under curve (*auc*) (**e** and **f**). *P < 0.05. *PU* perfusion units

Baseline skin endothelial blood flow in the forearm area was similar in patients with mottled and non-mottled skin (Figs. 1a and 2a). However, in the knee area, baseline skin blood flow was significantly lower in the mottled knee area as compared to non-mottled forearm skin (4 (4–6) vs. 7 (6–8) units, *P* < 0.05) (Figs. 1b and 2b).

### Regional endothelial function assessment

**Fig. 3:**
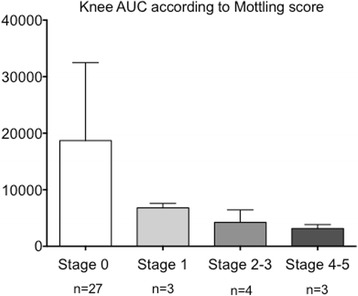
Analysis of skin endothelial function in the knee area according to mottling score. Skin blood flow was quantified after acetylcholine iontophoresis. *P* = 0.02, Kruskal-Wallis test. *AU*C area under curve

Ach iontophoresis was performed 6 (6–7) h after admission for sepsis patients and 7 (6–8) h after admission for septic shock patients. In response to iontophoresis of acetylcholine, we observed an increase in skin blood flow related to small vessel relaxation. In the forearm area, the increased skin blood flow (both peak value and area under the curve (AUC)) was similar in patients with mottled skin as compared to patients without mottled skin (Figs. 1a and 2c and e). However, the increase in skin blood flow in the knee area was significantly smaller in patients with mottled skin as compared to patients without mottled skin (peak value 7 (4–15) vs. 17 (8–50) units and AUC 3280 (2643–6440) vs. 7980 (4233–19,707), both *P* < 0.05) (Figs. 1b and 2d and f). We obtained the results with data expressed as variations (percentage) of skin blood flow induced by Ach iontophoresis relative to the individual baseline blood flow (Additional file 5A). Finally, we observed that the endothelial response to Ach declined with increase in the mottling score (Kruskal-Wallis test, *P* = 0.02) (Fig. 3).

### Skin microcirculatory assessment according to the outcome at 14 days

**Fig. 4:**
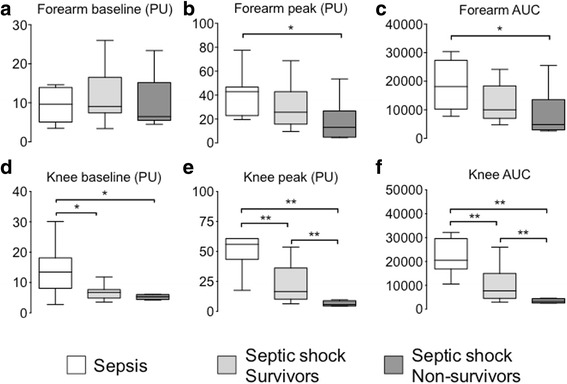
Analysis of skin microcirculatory blood flow measured in three groups of patients: patient with sepsis (n = 11), 14-day survivors with septic shock (n = 18), and 14-day non-survivors with septic shock (n = 8). Skin blood flow was quantified on the forearm and the knee area at baseline (**a**, **d**) and after acetylcholine iontophoresis (peak value (**b** and **e**) and area under the curve (*AUC*) (**c** and **f**). *P < 0.05, **P < 0.01. *PU* perfusion units

**Fig. 5:**
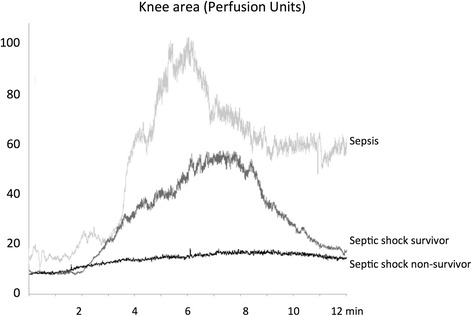
Examples of skin microcirculatory blood flow measurements in the knee area in three patients (one with sepsis, one 14-day survivor with septic shock, and one non-survivor with septic shock) on the forearm and the knee area at baseline and after acetylcholine iontophoresis

The relationship between skin endothelial function and outcome at 14 days is presented in Fig. 4. Overall, we observed a significant reduction in skin blood flow in the knee area in septic shock patients as compared to patients with sepsis (Figs. 4a and 5). The increase in skin blood flow following iontophoresis of Ach was impaired (both peak and AUC) in the knee area in patients with septic shock as compared to patients with sepsis. In patients with septic shock, the increase in blood flow was significantly smaller in non-survivors as compared to survivors (peak 6 (4–9)] vs. 16 (10–36) units, AUC 3256 (2600–4426) vs. 7704 (4539–15,011)], *P* < 0.01) (Fig. 4b, c and Fig. 5).

There was no difference in baseline skin blood flow in the forearm area between patients with sepsis and survivors of septic shock, nor was there any difference between survivors and non-survivors of septic shock (Fig. 4d). The increase in skin blood flow following iontophoresis of Ach in the forearm was significantly greater in patients with sepsis as compared to non-survivors of septic shock, but was similar to that in survivors of septic shock (Fig. 4e and f). There was no significant difference between survivors and non-survivors of septic shock. We obtained the results with data expressed as variations (percentage) of skin blood flow induced by Ach iontophoresis relative to the individual baseline blood flow (Additional file 5B).

## Discussion

Using validated non-invasive technology comprising laser Doppler flowmetry coupled with Ach iontophoresis, we demonstrated heterogeneous skin endothelial dysfunction in critically ill patients with severe infections. We found that the endothelium-dependent vasodilation was impaired in the mottled skin as compared to non-mottled skin. In addition, we found that the knee skin endothelial dysfunction was related to sepsis severity and 14-day mortality. Results were consistent across comparisons of blood flow or variations in blood flow induced by Ach iontophoresis.

Young et al. previously measured skin blood flow in healthy volunteers, patients with sepsis and patients recovering from coronary artery bypass grafting with a laser Doppler flowmeter. The authors did not find any baseline difference in skin blood flow between groups in the forearm area [27]. Here, we found a specific reduction in skin blood flow in the knee area in mottled vs. non-mottled skin. However, the presence of mottling in the knee area did not affect baseline skin perfusion in other areas such as the forearm skin, where mottling almost never develops.

Several experimental and clinical studies in sepsis have shown attenuated or lost endothelial cell response to chemical or physical stimulation, either in vivo or in isolated vessels [28]. Skin endothelial dysfunction has been previously reported in both chronic and acute vascular injury such as diabetes mellitus [29] or preeclampsia [30]. In sepsis patients, vascular dysfunction measured by peripheral arterial tonometry was reported [31]. However, this method that computed the changes in digital pulse volume amplitude during post-occlusive reactive hyperemia did not specifically evaluate microvascular endothelial function [32]. In our study, we documented skin endothelial dysfunction in skin-resistive small vessels in mottled areas of the knee. Endothelial dysfunction was more pronounced in patients with extensive mottling.

Skin endothelium function was evaluated at the same time in the knee and the forearm areas and we identified heterogeneous skin endothelial dysfunction in patients with sepsis. Spatial endothelial heterogeneity after septic injury has been previously reported in other organs. In animals, Morin et al. have analyzed endothelial inducible NO synthase (iNOS) messenger RNA (mRNA) expression in different gut compartments following endotoxinemia. The authors reported that iNOS mRNA expression was variable along the digestive tract, being marked expressed in the ileum, but weakly detected in the jejunum and colon. After lipopolysaccharide (LPS) injection, expression of iNOS mRNA was upregulated in both villus and crypt cells, although iNOS mRNA expression was more prominent in the former than the latter cell type [5]. Here, we reported a specific alteration in endothelial function in mottled knee skin that is probably related to impaired production of NO [19].

We found that knee skin endothelial dysfunction was more important in patients with septic shock as compared to patients with sepsis, and we also observed greater endothelial dysfunction in septic shock non-survivors as compared to survivors. Davis et al. also reported greater endothelial dysfunction in sepsis among patients with organ failure as compared to patients without organ failure [31].

When compared to patients with sepsis, skin blood flow response to Ach in the forearm area, tended to be smaller in septic shock survivors but was significantly reduced in septic shock non-survivors. This observation suggests that endothelial function was also altered in the forearm skin area but to a lesser extent when compared to the knee area. This less significant difference could also be partly explained by limited power due to the small number of included patients.

This observational study showed marked alteration in the increased blood flow response to Ach specifically in mottled skin, suggesting that mottling could be used as a reliable clinical indicator of endothelial dysfunction. We can speculate that in sepsis, patients with mottling would be the population of choice to test new microcirculatory targeting strategies.

Regarding confounding factors, we did not found any significant difference in baseline skin temperature in the forearm and knee areas. Norepinephrine doses were significantly higher in patients with septic shock who died. However, the endothelial function in forearm skin did not differ between survivors and non-survivors, suggesting that vasopressor doses were not directly responsible for the difference between the two groups in the skin endothelial response in the knee area.

## Conclusion

This is the first demonstration that mottling is associated with skin regional endothelial dysfunction in septic shock. Endothelial dysfunction in the knee skin area was more pronounced in 14-day non-survivors of septic shock when compared to survivors. These findings suggest that mottling could be used as a reliable clinical indicator of endothelial dysfunction.

## Keys messages

- There is endothelial dysfunction in the area of skin mottling in patients with sepsis
- Skin endothelial dysfunction is more pronounced in non-survivors of septic shock when compared to survivors

## Additional files

Additional file 1: Typical recording of endothelial function assessment in the forearm area (of a researcher) using laser Doppler flowmetry (LDF) and iontophoretic acetylcholine. (TIFF 1521 kb)

Additional file 2: Example of the recording of skin blood flow recorded on a healthy subject by laser Doppler flowmetry during three successive sessions of transdermal iontophoresis of acetylcholine (0.12 mA for 12 s each), separated by periods of 60s. *PU* perfusion unit. (TIFF 1521 kb)

Additional file 3: Baseline characteristics of patients. (DOCX 16 kb)

Additional file 4: Hemodynamic and tissue perfusion parameters of patients according to the presence of mottling. (DOCX 57 kb)

Additional file 5: Skin blood flow variations induced by Ach iontophoresis expressed relative to the individual baseline blood flow. a Skin blood flow variations according to the presence of mottling. b Skin blood flow variations according to the 14-day outcome. *P < 0.05, **P < 0.01. (TIFF 1521 kb)

## Abbreviations

Ach: Acetylcholine
AUC: Area under the curve
HR: Heart rate
ICU: Intensive care unit
iNOS: Inducible nitric oxide synthase
MAP: Mean arterial pressure
mRNA: Messenger RNA, NO, Nitric oxide
SAPS II: Simplified Acute Physiologic Score II
SOFA: Sequential Organ Failure Assessment
