# Supplementary figures and images for: Marked regional endothelial dysfunction in mottled skin area in patients with severe infections
Source: Crit Care. 2017 Jun 23;21:155. doi: 10.1186/s13054-017-1742-x (PMC5481873; doi:10.1186/s13054-017-1742-x)

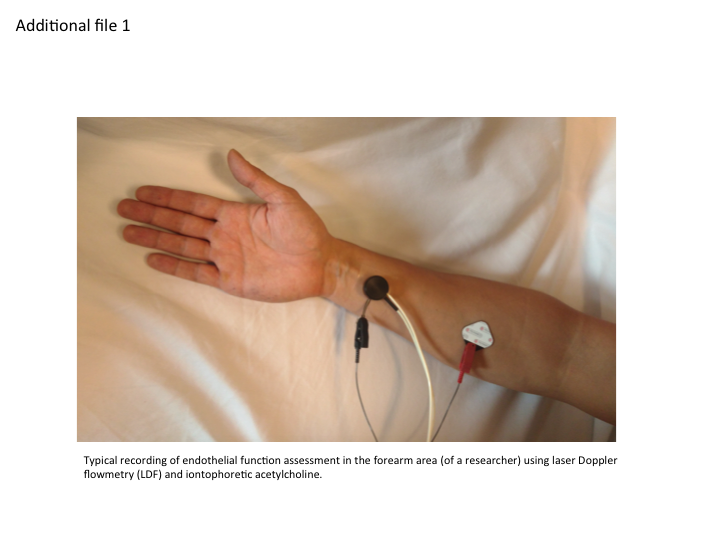

Supplement: Supplementary file 1 — Typical recording of endothelial function assessment in the forearm area (of a researcher) using laser Doppler flowmetry (LDF) and iontophoretic acetylcholine. (TIFF 1521 kb) [file 13054_2017_1742_MOESM1_ESM.tiff]

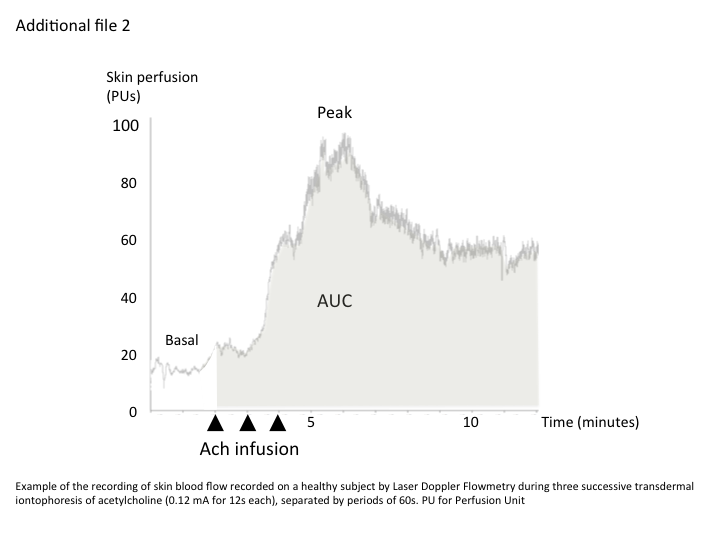

Supplement: Supplementary file 2 — Example of the recording of skin blood flow recorded on a healthy subject by laser Doppler flowmetry during three successive sessions of transdermal iontophoresis of acetylcholine (0.12 mA for 12 s each), separated by periods of 60s. PU perfusion unit. (TIFF 1521 kb) [file 13054_2017_1742_MOESM2_ESM.tiff]

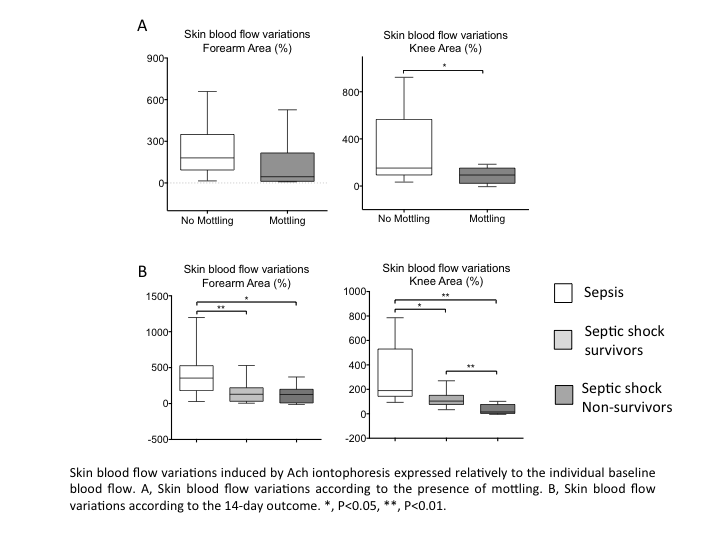

Supplement: Supplementary file 5 — Skin blood flow variations induced by Ach iontophoresis expressed relative to the individual baseline blood flow. a Skin blood flow variations according to the presence of mottling. b Skin blood flow variations according to the 14-day outcome. *P < 0.05, **P < 0.01. (TIFF 1521 kb) [file 13054_2017_1742_MOESM5_ESM.tiff]
