# Supplementary material for: Marked regional endothelial dysfunction in mottled skin area in patients with severe infections
Source: Crit Care. 2017 Jun 23;21:155. doi: 10.1186/s13054-017-1742-x (PMC5481873; doi:10.1186/s13054-017-1742-x)
Supplement: Supplementary file 3 — Baseline characteristics of patients. (DOCX 16 kb) [file 13054_2017_1742_MOESM3_ESM.docx]

**Additional file 3. Baseline characteristics of patients**

| **Characteristics** | **All patients** |
| --- | --- |
| Sepsis/Septic shock (n) | 11/26 |
| Age (years), median [IQR1; IQR3] | 59 [49; 68] |
| Male gender, (M/F) | 25/12 |
| Primary site of infection (n)  Lung  Abdomen  Urinary tract  Soft tissue  Other | 19  12  2  1  3 |
| SAPS II, median [IQR1; IQR3] | 46 [37; 72] |
| Mechanical ventilation (n, %) | 15 (48) |
| Norepinephrine (septic shock)  Dose [μg/kg/min] | 0.50 [0.17; 1.10] |

SAPS II was recorded at H24, Simplified Acute Physiology Score. Data are expressed as number and percentage or median and interquartile ranges (IQR).
