# Supplementary material for: Marked regional endothelial dysfunction in mottled skin area in patients with severe infections
Source: Crit Care. 2017 Jun 23;21:155. doi: 10.1186/s13054-017-1742-x (PMC5481873; doi:10.1186/s13054-017-1742-x)
Supplement: Supplementary file 4 — Hemodynamic and tissue perfusion parameters of patients according to the presence of mottling. (DOCX 57 kb) [file 13054_2017_1742_MOESM4_ESM.docx]

| **Hemodynamic parameters at H6** | **No mottling** | **Mottling** | **P-value** |
| --- | --- | --- | --- |
| N | 27 | 10 | - |
| Norepinephrine dose [μg/kg/min] | 0.10 [0; 0.30] | 0.80 [0.40; 1.20] | **0.003** |
| MAP (mmHg) | 73 [66; 82] | 70 [65; 78] | ns |
| Cardiac index (L/min/m^2^) | 2.7 [2.3; 3.1] | 2.5 [2.4; 3.2] | ns |
| Urinary output (mL/kg/h) | 0.75 [0.53; 1.54] | 0.53 [0.17; 1.10] | ns |
| Lactate level (mmol/L) | 1.4 [0.9; 2.2] | 2.3 [1.5; 7.2] | 0.07 |

**Additional file 4 : Hemodynamic and tissue perfusion parameters of patients according to the presence of mottling.** MAP, Mean Arterial Pressure; ns, non-significant. Data are expressed as median and interquartile ranges (IQR1-IQR3). Comparisons were done using a non-parametric Mann-Whitney test.
